# Formyl Peptide Receptors 1 and 2: Essential for Immunomodulation of Crotoxin in Human Macrophages, Unrelated to Cellular Entry

**DOI:** 10.3390/cells14151159

**Published:** 2025-07-26

**Authors:** Luciana de Araújo Pimenta, Ellen Emi Kato, Ana Claudia Martins Sobral, Evandro Luiz Duarte, Maria Teresa Moura Lamy, Kerly Fernanda Mesquita Pasqualoto, Sandra Coccuzzo Sampaio

**Affiliations:** 1 Laboratory of Pathophysiology, Butantan Institute, Sao Paulo 05503-900, Brazil; luciana.pimenta@fundacaobutantan.org.br (L.d.A.P.); ellen.kato@butantan.gov.br (E.E.K.); 2 Department of Pharmacology, University of Sao Paulo, Sao Paulo 05508-900, Brazil; 3 Center for Contaminants, Adolfo Lutz Institute, Sao Paulo 01246-000, Brazil; 4 Institute of Physics, University of Sao Paulo, Sao Paulo 05508-090, Brazil; elduarte@usp.br (E.L.D.); mtlamy@usp.br (M.T.M.L.); 5 LIM-14, Faculty of Medicine, University of Sao Paulo, Sao Paulo 05508-000, Brazil; kerlypasqualoto@gmail.com; 6 Department of Immunology, University of Sao Paulo, Sao Paulo 05508-900, Brazil

**Keywords:** crotoxin, formyl peptides receptors, macrophages, immunomodulation, internalization, in silico study

## Abstract

Crotoxin (CTX), the main toxin in *Crotalus durissus terrificus* venom, is a heterodimeric complex known for its antitumoral, anti-inflammatory, and immunomodulatory properties. In macrophages, CTX stimulates energy metabolism, pro-inflammatory cytokines, superoxide production, and lipoxin A_4_ secretion while inhibiting macrophage spreading and phagocytosis. These effects are completely blocked by Boc-2, a selective formyl peptide receptors (FPRs) antagonist. Despite the correlation between FPRs and CTX-mediated effects, their involvement in mediating CTX entry into macrophages remains unclear. This study aimed to investigate the involvement of FPRs in CTX entry into monocytes and macrophages. For this, THP-1 cells were silenced for FPRs or treated with Boc-2. Results demonstrated that FPR-related signaling pathways, which influence macrophage functions such as ROS release, phagocytosis, and spreading, were reduced in FPR-silenced cells. However, even in the absence of FPRs, CTX was efficiently internalized by macrophages. These findings suggest that FPRs are essential for the immunomodulatory effects of CTX, but are not involved in CTX internalization.

## 1. Introduction

Despite their toxicity, several studies have demonstrated the potential of compounds derived from snake venoms in treating various pathophysiological processes. Millions of years of evolution have given these substances specificity and selectivity toward targets, such as ion channels, receptors, enzymes, cell membranes, or metabolic pathways [1,2,3]. For instance, early studies using homeopathic doses of *Crotalus durissus terrificus* venom (CdtV) were initiated in patients with pain, mainly of neoplastic origin [3,4].

*Crotalus* (family *Viperidae*), known as rattlesnakes, are responsible for snakebites in Brazil, mainly caused by *Crotalus durissus terrificus*. Although systemic effects are significant, local inflammatory reactions at the bite site are typically minimal or absent [4,5]. The venom is composed of several bioactive toxins, including crotoxin, crotamine, convulxin, and gyroxin, with crotoxin (CTX) being the major component, constituting about 60% of the total venom [6,7].

CTX was first isolated and crystallized in 1938 by Slotta and Fraenkel-Conrat [8]; it is a heterodimeric β-neurotoxin comprising two subunits: Crotoxin A (CA), also known as crotapotin, and Crotoxin B (CB), a phospholipase A_2_ (PLA_2_) [9]. Although its pharmaco-kinetics and systemic effects have been partially studied, demonstrating rapid distribution and clearance from organs like the kidneys, spleen, and brain [10], the molecular mechanisms involved in its interaction with tissues and cells remain less understood.

Experimental studies have shown that CTX modulates inflammatory and immune responses [11,12] and exhibits antitumor, antiangiogenic, and immunomodulatory effects in both *in vivo* and *in vitro* models [13,14,15,16,17]. Formyl peptide receptors (FPRs) are G-protein-coupled receptors (GPCRs) widely expressed on macrophages, playing a key role in both innate and adaptive immunity [18,19]. Previous studies have demonstrated that CTX increases plasma concentrations of lipoxin A4 (LXA_4_) and its stable analog 15-epi-LXA4, which inhibit tumor growth and angiogenesis as well as stimulating macrophage secretory activity. Both studies indicate the involvement of FPRs [13,16,17]. Additionally, FPRs antagonists, such as Boc-1 and Boc-2, have been shown to partially or completely inhibit the effects of CTX on macrophages [16,17].

Elucidating the molecular recognition of CTX by macrophages is crucial to under-standing the mechanisms through which this toxin modulates cellular metabolism and function. While studies have documented CTX interactions with FPRs, the exact mechanisms and interaction sites on the cell membrane remain undefined. Therefore, this study aims to elucidate the specific interactions of CTX with membrane structures, focusing on FPR1 and FPR2 receptors, in order to deepen insights into their biological activities and evaluate the contribution of these receptors to CTX entry into macrophages.

## 2. Materials and Methods

### 2.1. CTX Purification

CdtV solution was subjected to anion-exchange chromatography as previously described by Rangel-Santos and collaborators [20], using a Mono-Q HR 5/5 column in an FPLC system (Pharmacia, Uppsala, Sweden). The fractions (1 mL/min) were eluted using a linear gradient of NaCl (0–1 mol/L in 50 mmol/L Tris-HCl, pH 7.0). Three peaks (p1, p2, and p3) were obtained: p2 corresponded to the pure CTX fraction (about 60% of the crude venom); peaks 1 and 3 included the other CdtV toxins. Before pooling, the fractions containing CTX were tested for homogeneity by non-reducing sodium dodecyl sulphate-polyacrylamide gel electrophoresis (12.5%) and the phospholipase A_2_ activity was assessed by a colorimetric assay using a synthetic chromogenic substrate as previous described [21].

### 2.2. THP-1 Cell Culture

Human monocyte THP-1 cell line was purchased from Rio de Janeiro Cell Bank (BCRJ, Duque de Caxias, Brazil; cell line code 0234) and is widely applied as an experimental model for in vitro studies of macrophage functions. Following the Peres and Curi [22] cell culture methodology, to ensure the homogeneity of morphophysiological characteristics, THP-1 cells were maintained in RPMI-1640 culture medium containing 10% fetal bovine serum (FBS), supplemented with 2-mercaptoethanol (50 μM) (Sigma Aldrich^®,^ St. Louis, MO, USA), sodium bicarbonate, NaHCO_3_ (20 mM), Hepes (10 mM), L-glutamine (2 mM). Cells were kept in 75 cm^2^ culture flasks (Corning^®^, Corning, NY, USA) in 5% CO_2_ incubator at 37 °C, with the replacement of the cell culture medium every 2–3 days.

### 2.3. Induction of Cell Differentiation

To promote the differentiation of THP-1 cells from monocytes to macrophages, phorbol myristate acetate ester (PMA) was used as an inducing agent. The differentiation protocol was modified based on previously established methods [23,24,25,26,27]. THP-1 cells were seeded at a cell density of 1 × 10^6^ cells/mL into 6-well plates and incubated with PMA (10 ng/mL) in RPMI medium for 12 h, at 37 °C and 5% CO_2_. After differentiation, THP-1 cells undergo significant changes in their morphology and behavior, adopting macrophage-like characteristics. Differentiated THP-1 cells were trypsinized and used in the subsequent assays.

### 2.4. Toxin Labeling Assay

Approximately 50 µg of purified toxin was suspended in 150 µL of 10 mM Hepes, pH 7.0. The pH of the Hepes buffer was also adjusted to 8.3 by the addition of sodium bicarbonate for further toxin washes. Fluorescein isothiocyanate (FITC, 250 µL) was added to the toxin solution. The reaction was conducted in the dark at room temperature for 3 h under continuous stirring and was stopped by the addition of 50 mM ammonium chloride. By using Vivaspin^®^ 10 kDa column (GE Healthcare; Little Chalfont, UK), the excess dye was washed with Hepes Buffer solution and the column was centrifuged. The conjugate CTX-FITC was collected after washing, leaving about 20 µL of labeled toxin at approximately 20 µg [28].

### 2.5. FPR1 SiRNA Transfection in THP-1 Cells

The technique applied was RNA silencing by electroporation, based on nucleofection technology. For this, THP-1 cells (monocytes, mϕ) and THP-1 cells differentiated into macrophages (Mϕ) as described previously were used. Cells were collected and prepared for nucleofection following the manufacture’s protocol (Lonza^®^, Basel, Switzerland). Human FPR1 siRNA, as described by the ThermoFisher Scientific^®^ (Waltham, MA, USA) datasheet, was then added to the cells. The sample was transferred to a specific cuvette for the 4D-NucleofactorTM System for cell electroporation. At the end of the process, the cuvette was washed with Phosphate Buffer Saline (PBS, pH 7.4) and the sample was transferred to culture plates. Expression can be detected 8 h after nucleofection. The silencing was confirmed by evaluating the expression of the receptors, using immunofluorescence (Section 2.7) and Western blotting (Section 2.9) techniques.

### 2.6. Assay Protocols and Pharmacological Treatments

To assess the role of FPRs in the entry of CTX in human monocytes/macrophages and to evaluate the effects of this toxin on cell function, THP-1 cells (monocytes, mϕ) or THP-1-derived macrophages (Mϕ) were silenced for formyl peptide receptors (mϕFPRs^−/−^ or MϕFPRs^−/−^) or left untransfected (mϕFPRs^+/+^ or MϕFPRs^+/+^). The cells were incubated with CTX at a concentration of 12.5 nM. In addition, under certain conditions, cells were pre-incubated with FPRs agonist fMLP (N-formyl-methionyl-leucyl-phenylalanine, Sigma Aldrich^®^) or FPRs antagonist Boc-2 (Boc-Phe-Leu-Phe-Leu-Phe, Phoenix Pharmaceuticals, Inc., Burlingame, CA, USA) before toxin exposure, according to the protocols below.

#### 2.6.1. CTX Treatment

THP-1 cells in different cell conditions (mϕFPRs^−/−^, MϕFPRs^−/−^, mϕFPRs^+/+^ and MϕFPRs^+/+^) were incubated in RPMI-1640 culture medium with CTX (12.5 nM, corresponding to 0.3 μg/mL) for 2 h at 37 °C, 5% CO_2_. This CTX concentration has been shown to significantly modulate macrophage metabolism and function, as reported in previous studies [16,17,29]. After incubation, cell culture medium was removed, cells were washed with PBS and fresh culture medium was added for the experiments.

#### 2.6.2. fMLP Treatment

THP-1 cells in different cell conditions, either silenced for formyl peptide receptors (mϕFPRs^−/−^ and MϕFPRs^−/−^) or untransfected (mϕFPRs^+/+^ and MϕFPRs^+/+^), were incubated and stimulated in the presence of fMLP, at a concentration of 10 μM for 2 h at 37 °C and 5% CO_2_. Afterward, the cells were washed with PBS and incubated in the presence of a fresh culture medium alone or with 12.5 nM CTX for the functional assays (Section 2.8).

#### 2.6.3. Boc-2 Treatment

THP-1 cells in different cell conditions, either silenced for formyl peptide receptors (mϕFPRs^−/−^ and MϕFPRs^−/−^) or untransfected (mϕFPRs^+/+^ and MϕFPRs^+/+^), were incubated and stimulated in the presence of Boc-2, at concentrations of 50 µM, 100 µM, and 150 µM [16], for 15 min. After the incubations, the cells were washed with PBS and incubated in the presence of a fresh culture medium alone or with CTX (12.5 nM), for 1 min, 2 h, and 24 h at 37 °C and 5% CO_2_ following immunofluorescence assay (Section 2.7).

### 2.7. Fluorescence Assay of THP-1 Cells Incubated with FITC-CTX

MϕFPRs^−/−^ and MϕFPRs^+/+^ (2.5 × 10^5^ cells/well) were seeded into 24-well plates and allowed to adhere for 2 h. Following the adhesion period, the cells were incubated with CTX-FITC at defined time intervals. After incubation, cells were washed with PBS followed by fixation and permeabilization steps. A fixative solution containing 4% paraformaldehyde (PFA) and 5% sucrose was added to each well and incubated for 15 min at room temperature. Following this, a permeabilizing solution containing 4% PFA, 5% Sucrose, and 0.5% Triton X-100 was added for 5 min. After the washing step, 70 µL/well of Rhodamine Phalloidin (1:500; Molecular Probes, Burlington, CA, USA) was added to stain actin filaments, and DAPI (4′,6′-diamidino-2-phenylindole) (Sigma Aldrich) was used for nuclear visualization. Both staining reagents were incubated for 30 min at room temperature. After an additional washing step, the coverslips were mounted with Vectashield^®^ (Vector Laboratories, Newark, CA, USA). Images were acquired using a using a Leica DMi8 confocal microscope (Leica Microsystems, Mannheim, Germany), equipped with a DFC 310 FX digital camera, 40× magnification with oil immersion. Images were captured with the Leica Application Suite Advanced Fluorescence (LAS AF) software, version 3.3. 

### 2.8. Evaluation of Functional Assays

#### 2.8.1. Measurement of Hydrogen Peroxide (H_2_O_2_) Production

Hydrogen peroxide production was quantified using the method described by Pick and Mizel, which is based on a horseradish peroxidase (HRP)-mediated conversion of phenol red into a colored compound in the presence of H_2_O_2_ [30]. Both mϕFPRs^−/−^ and mϕFPRs^+/+^ (4 × 10^5^/well) were seeded into 96-well plates and incubated for 24 h. After this period, cells were washed with PBS and incubated with CTX (12.5 nM) for 2 h in RPMI 1640 medium, at 37 °C, 5% CO_2_. Following CTX treatment, cells were washed again and incubated for 1 h with 100 µL of phenol red solution (PRS) per well. For the basal condition, cells received PRS alone; for the stimulated condition, PRS was supplemented with PMA (10 ng/mL). The PRS consisted of 140 mM NaCl, 10 mM potassium phosphate buffer (pH 7.0), 5.5 mM dextrose, 0.28 mM phenol red, and 8.5 U/mL of HRP. The reaction was stopped by the addition of 1 N sodium hydroxide (NaOH). Absorbance was measured at 620 nm using a spectrophotometer. The assay was performed in quadruplicate, and results were expressed as nanomoles of H_2_O_2_ per 4 × 10^5^ cells as described in [16].

#### 2.8.2. Macrophage Spreading Capacity

After pharmacological treatment with fMLP, MϕFPRs^−/−^ and MϕFPRs^+/+^ (2.5 × 10^5^ cells) were seeded onto glass coverslips in 24-well plates and incubated in RPMI-1640 culture medium in serum-free condition, with 12.5 nM CTX, for 2 h. After this period, cells were washed with PBS and cultured for 4 h in RPMI 1640 medium serum free FBS, at 37 °C, 5% CO_2_. Then, the coverslips were washed, stained, and fixed in Rosenfeld’s solution. The coverslips were then mounted onto slides. The percentage of spread cells (characterized by a flattened morphology with extended cytoskeleton projections and strong adhesion to the substrate) and non-spread cells was determined for each sample of 100 cells. Images were acquired using a Leica DFC420 light microscope (Leica Microsystems, Wetzlar, Germany).

#### 2.8.3. Phagocytosis Assay

After pharmacological treatment with fMLP, MϕFPRs^−/−^ and MϕFPRs^+/+^ (2.5 × 10^5^ cells) were treated or not with CTX at a concentration of 12.5 nM, for 2 h. After 4 h of the spreading assay (as described in the item 2.8.2), the cells were incubated in RPMI 1640 culture medium serum free, containing zymosan particles in a 10:1 ratio of cells and incubated for 1 h. After this, the coverslips were washed in PBS and stained with Rosenfeld solution. The coverslips were mounted on slides, using Entellan^®^. Phagocytosis was quantified by analyzing 100 randomly selected cells per coverslip under light microscopy. The phagocytic score was determined based on the proportion of cells that had internalized 0, 1, 2, or ≥3 zymosan particles. Images were acquired using a Leica DFC420 light microscope (Leica Microsystems, Germany).

### 2.9. Expression of Formyl Peptide Receptors and Signaling Pathway

MϕFPRs^−/−^ and MϕFPRs^+/+^ (1 × 10^6^) were lysed in RIPA buffer (Sigma Aldrich, R0278) containing protease and phosphatase inhibitors (Sigma Aldrich^®^, P0044; P5726; P8340). After sample centrifugation (16,000× *g*, 30 min, 4 °C), the supernatants were collected, and protein concentration was quantified using the BCA (bicinconinic acid) assay. Protein extracts (30 µg) were denatured, separated by SDS-PAGE, and transferred to a nitrocellulose membrane. Membranes were blocked in TBS-T containing 5% BSA for 1 h and incubated overnight at 4 °C with the following primary antibodies (1:1000 dilution in TBS-T + 5% BSA): anti-FPR1 (ab113531), anti-Rac1 (ab203129), anti-SRC (ab40660), and anti-PI3K p85 (ab40776). Anti-GAPDH (ab9485) was used as the loading control. After overnight incubation, membranes were washed and incubated for 2 h at room temperature with HRP-conjugated secondary antibodies (1:10.000 dilution in TBS-T + 5% BSA): goat anti-rabbit IgG (ab6721) and goat anti-mouse IgG (ab205719). All primary and secondary antibodies were purchased from Abcam^®^ (Cambridge, UK). Protein detection was visualized by an enhanced chemiluminescence kit (Thermo Scientific^®^), and images were acquired using a photo documentation system (Alliance 9.7, UVITEC^®^, Cambridge, UK). Quantitative analysis of protein expression was measured with Image J software version 1.53 (National Institutes of Health).

### 2.10. In Silico Study

Based on the well-established CTX structure, the fragments of each subunit were mapped and distributed in databases to identify compounds with similar structure to CTX, particularly those interacting with G-protein-coupled receptors. Protein Data Bank (PDB) (https://www.rcsb.org/ (accessed on 9 January 2020)) [31] identified FASTA sequences and three-dimensional structures of the CTX subunits. After identification, a database search was performed using DrugBank 5.1.0 (http://www.drugbank.ca (accessed on 22 February 2021)) [32] and Therapeutic Targets Database (TTD) (https://idrblab.net/ttd/ (accessed on 1 January 2018)) [33], to assess the chemical similarity with the substances under investigation or commercially available drugs, and the respective molecular targets identified and validated for the compounds and drugs deposited in the database. A search for chemical structure was performed by prioritizing similarity at the 0.7 threshold, without specifying maximum or minimum weight, and including both approved and experimental compounds. Therapeutic Targets Database (TTD) is a database that provides information on known and explored therapeutic and protein nucleic acid targets, the target disease, pathway information, and the corresponding drugs targeted at each of these targets [33]. The degree of similarity of these identified targets was assessed by the BLAST program (Version 2.9), with the identified targets listed by their e-value (from lowest to highest). The sequences of the drug targets like CTX structure were obtained from TTD and compared to type I formyl peptide (FPR1). All e-values were considered when comparing FPR sequences with receptors found in the Drug Bank.

### 2.11. Statistical Analysis

Values will be expressed as mean ± SEM (standard error of the mean) and analyzed statistically by ANOVA and the Tukey–Kramer test (INSTAT—GraphPad Software, version 3.01), comparing the values of the control group with the values of the experimental groups. The differences will be considered significant for the level of *p* < 0.05 (5%).

## 3. Results

### 3.1. Differentiation of TPH-1 Cells into Macrophages

In this study, after inducing the differentiation of THP-1 monocytes into macrophages, using PMA, cell morphology was assessed. After differentiation, THP-1 cells undergo significant changes in their morphology and behavior, adopting macrophage-like characteristics (Figure S1B). The main changes include strong adherence to the substrate, in contrast to the undifferentiated state in which they remain in suspension (Figure S1A); they become larger with a more flattened and spread-out shape, typical of macrophages, and cytoskeletal rearrangement also occurs, with the formation of structures such as lamellipodia, which enhances their phagocytic capacity, a functional marker of macrophages. In both protocols, the cells showed viability above 98%, evaluated by the trypan blue exclusion test.

### 3.2. Effectiveness of FPR1 Silencing in Differentiated and Undifferentiated THP-1 Cells

**Figure 1:**
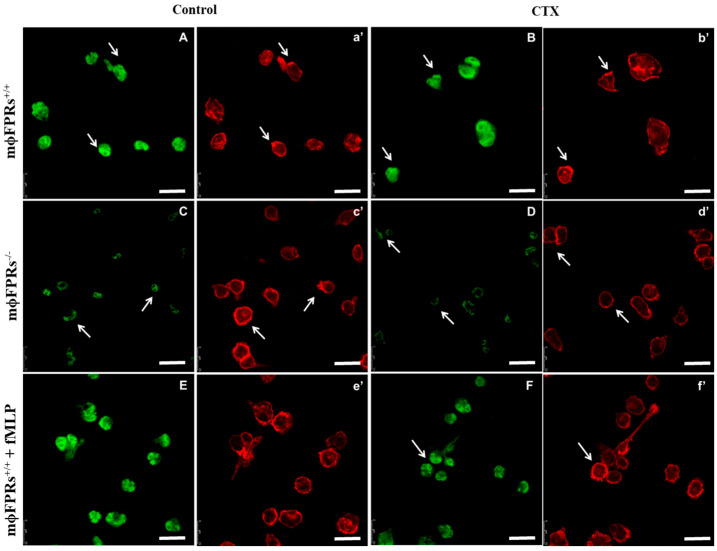
THP-1 monocytes with (mϕFPRs^−/−^) or without (mϕFPR^+/+^) FPR silencing in different treatments. THP-1 monocytes were incubated with CTX for 2 h and fixed and stained for formyl peptide receptors (FITC, green) and for actin cytoskeleton (rhodamine phalloidin, red). (**A** and **a’**) mϕFPR^+/+^ in RPMI medium, (**B** and **b’**) mϕFPR^+/+^ treated with CTX (12.5 nM), (**C** and **c’**) mϕFPRs^−/−^ in RPMI medium, (**D** and **d’**) mϕFPRs^−/−^ treated with CTX (12.5 nM), (**E** and **e’**) mϕFPR^+/+^ stimulated with fMLP, (**F** and **f’**) mϕFPR^+/+^ stimulated with fMLP and treated with CTX. White arrows indicate the colocalization of FPR1 with cortical actin. Scale Bar = 25 µm.

**Figure 2:**
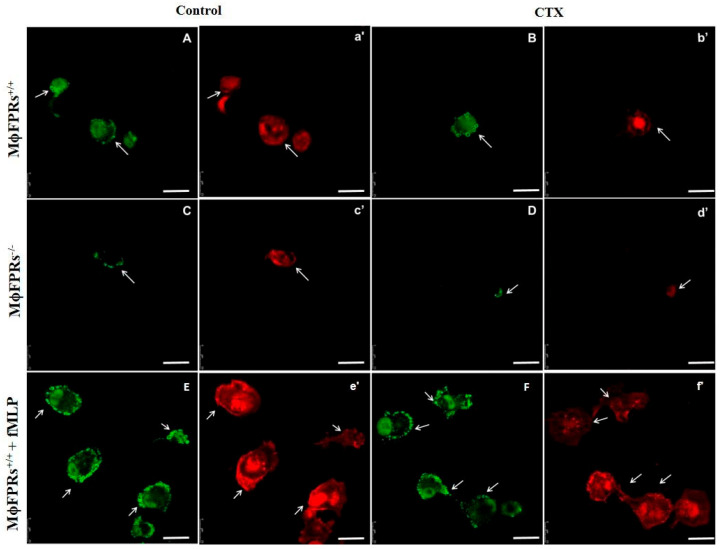
THP-1-differentiated macrophages with (MϕFPRs^−/−^) or without (MϕFPR^+/+^) FPR silencing in different treatments. THP-1 cells differentiated into macrophages were incubated with CTX and fixed and stained for formyl peptide receptors (FITC, green) and for actin cytoskeleton (rhodamine phalloidin, red). (**A** and **a´**) MϕFPR^+/+^ in RPMI medium, (**B** and **b´**) MϕFPR^+/+^ treated with CTX (12.5 nM), (**C** and **c´**) MϕFPRs^−/−^ in RPMI medium, (**D** and **d´**) MϕFPRs^−/−^ treated with CTX (12.5 nM), (**E** and **e´**) MϕFPR^+/+^ stimulated with fMLP, (**F** and **f´**) MϕFPR^+/+^ stimulated with fMLP and treated with CTX. White arrows indicate the colocalization of FPR1 with cortical actin. Scale Bar = 25 µm.

To confirm FPR silencing in THP-1 cells (mϕ) or THP-1-differentiated macrophages (Mϕ), immunofluorescence staining was performed. Figure 1 and Figure 2 (panels C and D) illustrate a decrease in the fluorescence of the FITC marker, indicating a reduction in FPR receptors in both monocytes and macrophages when compared to the Control and CTX-groups (Figure 1 and Figure 2, panels A and B, respectively). Additionally, we performed rhodamine phalloidin staining to visualize the actin filaments within the cells. Notably, treatment with CTX and fMLP led to an increase in FPR receptor expression. It is important to highlight that all images for this experiment were captured using the same gain settings, ensuring that the observed reduction accurately reflects the decrease in receptor availability rather than variations in microscope fluorescence intensity.

### 3.3. Evaluation of Functions of THP-1-Differentiated Macrophages

**Figure 3:**
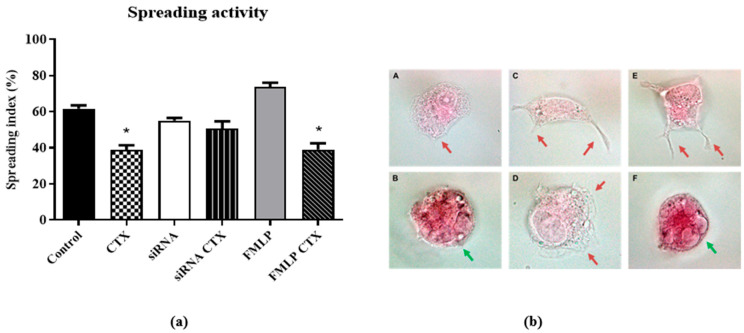
Effect of CTX on spreading activity in THP-1-differentiated macrophages. In (**a**): Representative graph showing the effect of CTX on spreading activity (%) in MϕFPRs^+/+^, MϕFPRs^−/−^, and MϕFPRs^+/+^ stimulated with fMLP groups. In (**b**): Representative spreading activity images corresponding to the graph above. (**A**) Control group; (**B**) CTX (12.5 nM); (**C**) MϕFPRs^−/−^; (**D**) MϕFPRs^−/−^ CTX-treated; (**E**) MϕFPRs^+/+^ stimulated with fMLP; (**F**) MϕFPRs^+/+^ CTX-treated. Red arrows indicate protrusions formed during cell spreading, corresponding to lamellipodia (anterior protrusions) and filopodia (posterior protrusions). Green arrows indicate intracellular vacuoles. Results are expressed as mean ± SEM for 3 samples per group and represent 3 independent assays. * *p* < 0.05 compared with the respective control groups. Images were acquired at 20× magnification.

Regarding the effects of CTX on cell spreading, THP-1-differentiated macrophages (adherent cells) treated with CTX demonstrated reduction in the spreading percentage by 35% when compared to the control (Figure 3a). The same effect was observed in the fMLP-stimulated group, where the inhibitory effect of CTX was more pronounced, reaching about 47% inhibition (Figure 3a). In the silenced groups, the percentages of spread cells were very similar, with no difference between the CTX-treated and control groups (Figure 3b(A,B)) for visualization in 20× objective microscope). Additionally, increased intracellular vacuoles were also observed, indicative of high metabolic activity induced by CTX (Figure 3b).

**Figure 4:**
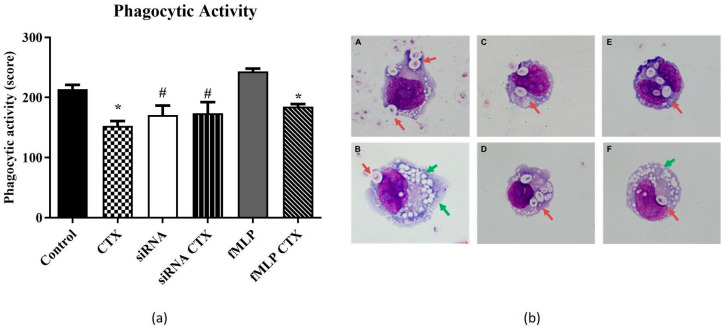
Representative graph of the effect of CTX in phagocytic activity by THP-1-differentiated macrophages. In (**a**), representative graph showing the effect of CTX on phagocytosis activity (score) in MϕFPRs^+/+^, MϕFPRs^−/−^, and MϕFPRs^+/+^ stimulated with fMLP groups. In (**b**), representative phagocytic activity images corresponding to the graph above. (**A**) Control group; (**B**) CTX (12.5 nM); (**C**) MϕFPRs^−/−^; (**D**) MϕFPRs^−/−^ CTX-treated; (**E**) MϕFPRs^+/+^ stimulated with fMLP; (**F**) MϕFPRs^+/+^ CTX-treated. The red arrows demonstrate the particles of zymosan phagocyted by the cells. Green arrows indicate intracellular vacuoles. Data are presented as mean ± SEM for 3 samples per group and represent 3 separate assays. * *p* < 0.05 compared with the respective control groups. ^#^ *p* < 0.05 compared to the fMLP-control group. Images were acquired at 20× magnification.

For phagocytosis activity, CTX reduced (29%) the number of phagocytosed particles by THP-1-differentiated macrophages compared to the control group (Figure 4b(A,B)). A similar inhibitory effect (24%) was observed in CTX-treated cells stimulated with fMLP (Figure 4b(F)). In the MϕFPRs^−/−^ group, no differences in phagocytic activity were observed between the control and CTX-treated groups (Figure 4b(C,D). Compared to the MϕFPRs^+/+^ control, a reduction in phagocytic activity was noted in the MϕFPRs^−/−^ group, although this difference was not statistically significant. However, a significant difference in phagocytic capacity was observed between the MϕFPRs^−/−^ and MϕFPRs^+/+^ control stimulated with fMLP group, showing an increase of approximately 42% in phagocytic activity (Figure 4b(E)).

**Figure 5:**
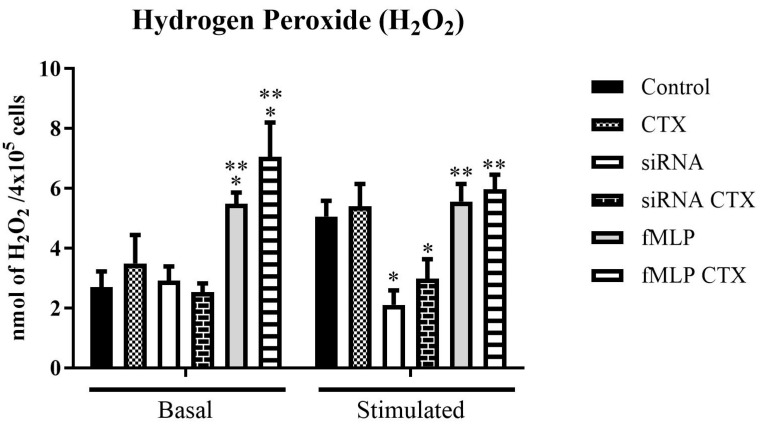
Effect of CTX in Hydrogen Peroxide (H_2_O_2_) production by THP-1 cells (mϕFPRs^+/+^ and MϕFPRs^+/+^). Cells were silenced and stimulated with fMLP under basal and PMA-stimulated conditions, while non-silenced and fMLP-untreated cells were used as control. The results are expressed as mean ± SEM for 3 samples per group, representing 3 independent experiments. * *p* < 0.05 compared to the respective control groups. ** *p* < 0.05 compared to the respective siRNA groups.

Additionally, results demonstrated increased H_2_O_2_ production in CTX-treated cells across different experimental groups, both under basal and PMA-stimulated conditions. In this context, basal condition refers to cells incubated with phenol red solution (PRS) alone, while the stimulated condition involves the use of PRS supplemented with PMA (10 ng/mL) to induce an oxidative response. Under basal condition, the mϕFPRs^+/+^ and MϕFPRs^+/+^ groups showed low H_2_O_2_ production, with no significant CTX activity observed. However, when compared to MϕFPRs^+/+^ stimulated with fMLP, H_2_O_2_ production was statistically increased in both the control and CTX-treated cells (Figure 5). Under PMA stimulation, CTX induced a slight increase in H_2_O_2_ production in the mϕFPRs^−/−^ group. Moreover, H_2_O_2_ production was similar between the mϕFPRs^+/+^ and MϕFPRs^+/+^ stimulated with fMLP groups, suggesting that the cells reach a plateau.

### 3.4. Formyl Peptide Receptor Expression and Activation

**Figure 6:**
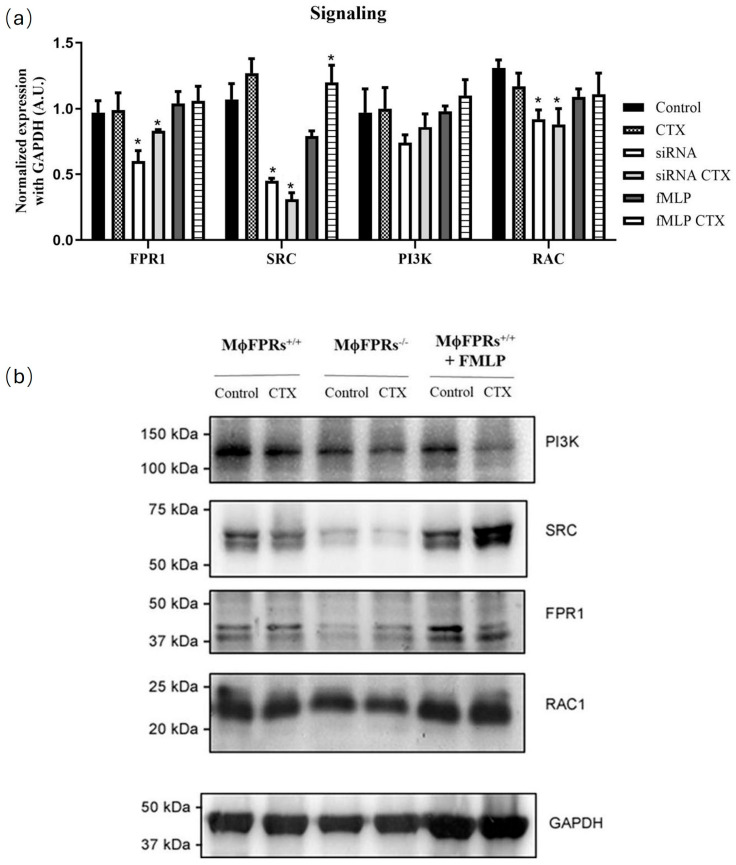
Effect of CTX on the expression of FPR1 and signaling proteins (RAC1, SRC, and PI3K). (**a**) Quantitative analysis of FPR1, RAC1, SRC, and PI3K protein expression normalized with GAPDH (loading control). (**b**) Representative Western blotting. Results are expressed as mean ± SEM for 3 samples per group and represent 3 independent experiments. * *p* < 0.05 compared with the respective control groups.

To investigate the effects of CTX on FPR expression and the FPR-signaling pathway, Western blotting was performed to detect protein expression of FPR1, PI3K, SRC, RAC1 in MϕFPRs^−/−^ cells (Figure 6a,b). Compared to control group of non-silenced THP-1 cells, FPR1 protein expression in MϕFPRs^−/−^ cells was reduced by 38%. In MϕFPRs^−/−^ cells, CTX slight increased FPR1 protein expression by 14% compared to its control. PI3K expression was similar between the non-silenced and fMLP-stimulated cells in both the control and CTX-treated group. However, in MϕFPRs^−/−^ cells, PI3K expression was reduced but no statistically significant differences were observed. In contrast, in both the MϕFPRs^−/−^ control and CTX-treated cells, Rac expression was reduced by 33% compared to the control MϕFPRs^+/+^ group and 19% compared to the control MϕFPRs^+/+^ stimulated with fMLP. Interestingly, SRC expression was markedly reduced by 58% in the MϕFPRs^−/−^ control cells and 71% in the MϕFPRs^−/−^ CTX-treated cells compared to the respective MϕFPRs^+/+^ group. SRC expression was highly expressed in the MϕFPRs^+/+^ stimulated with fMLP treated with CTX, showing a 51% increase compared to its control (Figure 6a,b).

### 3.5. Membrane Permeability Experiment—Fluorescent Marking-CTX

**Figure 7:**
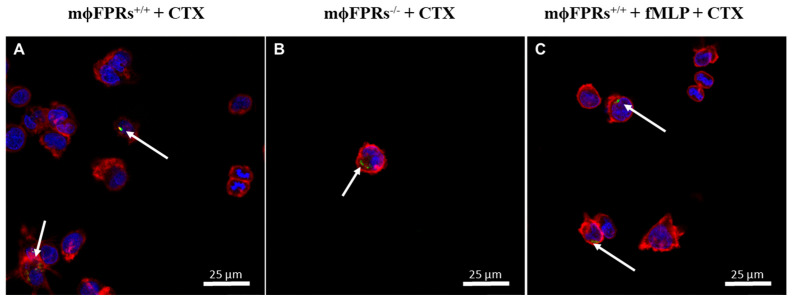
CTX-FITC internalization in THP-1 monocytes. Immunofluorescence imaging of THP-1 undifferentiated cells after 2 h incubation with CTX-FITC (green). (**A**) Control group (mϕFPRs^+/+^), (**B**) FPR-silenced group (mϕFPRs^−/−^), (**C**) fMLP-stimulated (mϕFPRs^+/+^ + fMLP). Actin filaments (red) and nuclear (blue) staining by rhodamine phalloidin and DAPI, respectively. White arrows highlight the cellular uptake of CTX-FITC. Scale bar: 25 µm.

**Figure 8:**
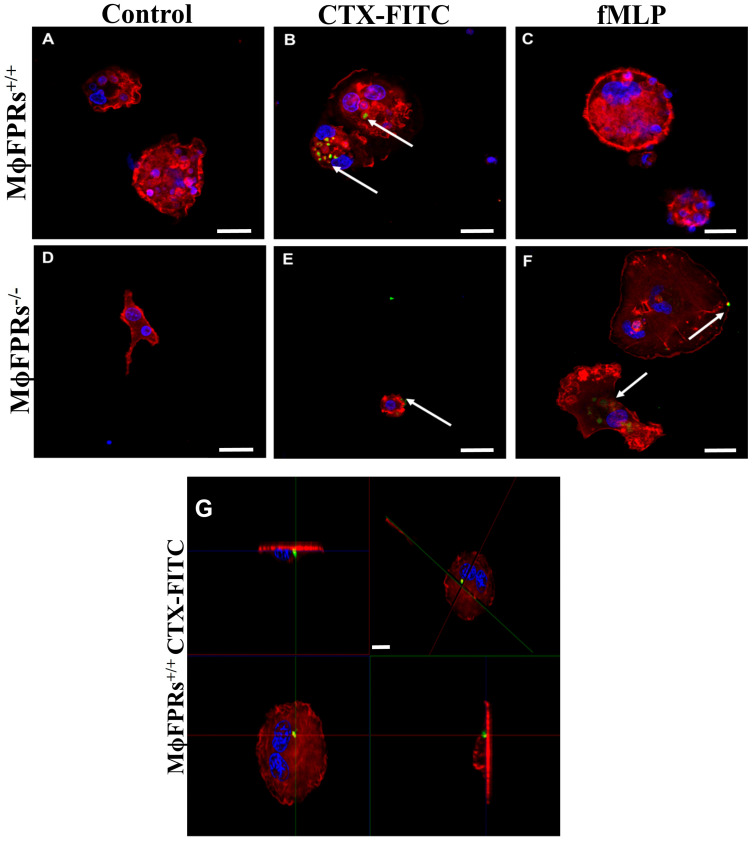
CTX-FITC internalization in THP-1-differentiated macrophages. Immunofluorescence imaging of THP-1-differentiated macrophages after 2 h incubation with CTX-FITC (green). (**A**) Control group with FITC alone; (**B**) control group with CTX-FITC; (**C**) fMLP-stimulated (MϕFPRs^+/+^ fMLP); (**D**) silenced group with FITC alone; (**E**) silenced group with CTX-FITC; (**F**) silenced group fMLP-stimulated; (**G**) orthogonal plane view of THP-1-differentiated macrophages with CTX-FITC. Actin filaments (red) and nuclear (blue) staining by rhodamine phalloidin and DAPI, respectively. White arrows highlight the cellular uptake of CTX-FITC. Scale bar: 25 µm.

Immunofluorescence analysis showed that CTX-FITC was internalized by both non-silenced (Figure 7A) and FPR-silenced THP-1 monocytes (Figure 7B), indicating that formyl peptide receptors are not required for CTX entry. Additionally, in the presence of the FRP agonist, fMLP, CTX-FITC was also detected inside the cells (Figure 7C), supporting this evidence. Similarly, CTX-FITC was also internalized on THP-1-differentiated macrophages, both non-silenced (Figure 8B) and silenced (Figure 8E). Figure 8G shows an orthogonal plane view of the CTX-FITC localization inside MϕFPRs^+/+^ cells. As a control group, cells were incubated with FITC without CTX conjugation, and no fluorescence was detected in either the silenced or non-silenced condition (Figure 8A,D). In the THP-1-differentiated macrophages stimulated with fMLP, CTX-FITC was not internalized; but in the FPR-silenced macrophages, CTX-FITC was internalized (Figure 8C,F).

**Figure 9:**
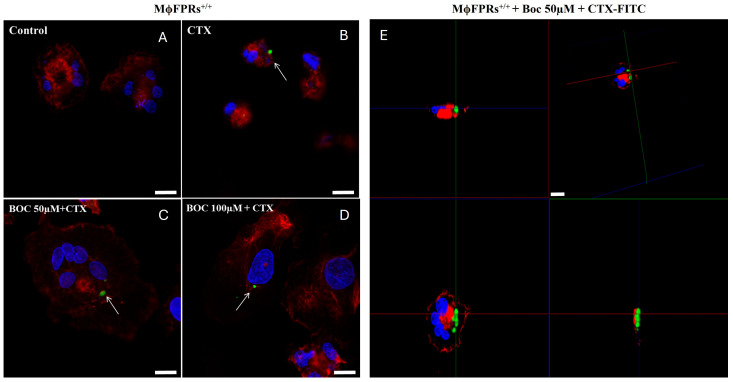
CTX-FITC internalization in THP-1-differentiated macrophages with FPR antagonist. Immunofluorescence imaging of THP-1-differentiated macrophages, previously incubated with Boc-2, followed by incubation with CTX-FITC (green) for 2 h. (**A**) MϕFPRs^+/+^ incubated with RPMI medium containing FITC (control), (**B**) CTX-FITC, or (**C**) MϕFPRs^+/+^ pre-incubated with 50 µM Boc-2 or (**D**) 100 µM Boc-2, then incubated with 12.5 nM CTX-FITC. (**E**) Orthogonal plane view of THP-1-differentiated macrophages pre-incubated with 50 µM Boc-2, followed by CTX-FITC incubation. Actin filaments (red) and nuclear (blue) staining by rhodamine phalloidin and DAPI, respectively. White arrows highlight the cellular uptake of CTX-FITC. Scale bar: 25 µm.

After blocking formyl peptide receptors with Boc-2 at concentrations of 50 µM or 100 µM, for 40 min, immunofluorescence imaging demonstrated that CTX-FITC was internalized into the THP-1-differenciated macrophages at both concentrations (Figure 9C,D), suggesting that FPRs are not essential for the toxin’s entry into the cells. As controls, cells exposed to FITC were used as a negative control (Figure 9A), while cells incubated with CTX-FITC served as a positive control (Figure 9B). Figure 9E shows an orthogonal plane view of the CTX-FITC localization inside MϕFPRs^+/+^ cells after Boc-2 incubation.

### 3.6. Analysis of DrugBank and TTD Database

Although Crotoxin was internalized into the monocytes and macrophages even with the blockade of the formyl peptide receptors, these data do not exclude the possibility that other G-protein-coupled receptors may be involved in the toxin/lipid bilayer process. Based on this, an in silico study was conducted using well-established databases to understand how known fragments might interact and whether they could be associated with other receptors. In the DrugBank database, there was a limitation on amino acid sequence length, making it impossible to use the whole CTX sequence. Based on the acquired data, four tables were generated containing approved or investigational drugs with structural similarities to CTX (subunits CA and CB). Selected compounds and drugs were added to the tables based on their similarity score to CTX or by indications similar to the actions already described for the toxin. In total, 27 results were obtained for the CA α chain, 11 results for the CA β chain, 12 results for the CA γ chain, and 61 results for CB. Among these, seven key compounds were highlighted for the CA α chain (Table S1), eight for the CA β chain (Table S2), eight for the CA γ chain (Table S3), and twenty-five results for CB (Table S4). Additionally, Table S5 shows the similarities of different targets found in the Drug Bank database in comparison to FPRs and FPR1, based on information from the TTD database.

## 4. Discussion

Formyl peptide receptors (FPRs) are seven-transmembrane G-protein-coupled receptors involved in host defense and inflammation. FPRs consist of three isoforms: FPR1, FPR2, and FPR3, which recognize peptides containing formylated methionine, derived from bacteria and mitochondrial proteins [34,35]. FPR1 and FPR2 are highly expressed on phagocytic cells while FPR3 is found in monocytes. One of the most potent ligands for human FPR1 is the *N*-formylmethionyl-leucyl-phenylalanine (fMLP), a peptide derived from *Escherichia coli*. In contrast, FPR2 is activated by endogenous peptides and proresolving lipids, such as Lipoxin A4. These FPR agonists activate multiple signaling pathways involved in inflammation, including chemotaxis, phagocytosis, superoxide production, degranulation, and transcriptional regulation [36,37,38]. Previous studies have demonstrated the essential role of FPRs in the immunomodulatory activities of CTX, particularly in macrophages. However, the correlation between these receptors and CTX internalization in macrophages has not yet been demonstrated. Therefore, the present study aimed to investigate whether the absence of FPRs on macrophages (MϕFPRs^−/−^) affects CTX entry, while simultaneously evaluating the effects on macrophage functions. For these evaluations, human THP-1 cell line was used to analyze the cell functions of both monocytes and macrophages.

It is well established in the literature that monocyte THP-1 cells can be differentiated into a macrophage-like phenotype using phorbol-12-myristate-13-acetate (PMA), 1α, 25-dihydroxyvitamin D3 (vD3), or macrophage colony-stimulating factor (M-CSF) [25,39,40]. After these stimuli, the cells acquire the ability to adhere and expand their cytoskeleton, characteristics that are considered key determinants of the differentiation of THP-1 monocytes into macrophages [23,36,41]. In this study, after promoting the differentiation of THP-1 cells from monocytes to macrophages using PMA as an inducer, the cells consistently remained viable with these characteristics.

To investigate the role of FPRs in CTX entry into macrophages, THP-1 cells were subjected to the mRNA silencing of FPR1. In parallel, pharmacological treatment was applied by pre-incubating the cells with Boc-2, an antagonist for FPR1 and FPR2, at concentrations of 50 µM or 100 µM. Transfection with siRNA or plasmid DNA is an efficient and widely used tool for studying macrophage functions. Although there are many different approaches available for transfecting cells, only a few methods are effective for efficient macrophage transfection [42,43]. The protocol used in this study provides a reliable method for transfecting human THP-1 macrophages and monocytes, ensuring cell viability as well as efficient transfection [43]. In our experimental model, the siRNA transfection was confirmed by immunofluorescence and Western blotting assays, in both monocytes and macrophages.

Sampaio and collaborators [29] demonstrated in in vivo models that CTX exerts a dual effect on macrophages. The toxin stimulates the production of reactive oxygen and nitrogen species (ROS and NOS, respectively), as well as lipid mediators such as lipoxin A4 (LXA_4_) and its stable analog 15-Epi-LXA_4_ while inhibiting cell spreading and phagocytic activity. In this study, THP-1-differentiated macrophages (MϕFPRs^+/+^) were subjected to cell spreading and phagocytosis assays. As observed in macrophages obtained from rodents [29], our results demonstrated that CTX reduces the spreading and phagocytosis functions of human macrophages. Interestingly, when MϕFPRs^+/+^ were stimulated with FPR agonist fMLP, these cell functions were significantly inhibited by CTX. However, in FPR-silenced macrophages (MϕFPRs^−/−^), CTX did not exert the same effect, presenting similar responses to its control group and the MϕFPRs^+/+^ group.

The measurement of H_2_O_2_ was possible in the THP-1 monocytes, whereas the THP-1-differentiated macrophages exhibited undetectable levels of H_2_O_2_. In the monocytic THP-1 cells, we observed a pattern consistent with the literature: an increase in H_2_O_2_ in cells treated with CTX and stimulated with PMA, across the different experimental groups. Costa and collaborators [16] demonstrated that CTX increased H_2_O_2_ release by macrophages; however, when pre-incubated with Boc-2 CTX, the stimulatory effect of CTX was abolished. Supporting these findings, our results demonstrated that in mϕFPRs^−/−^ cells, the production of H_2_O_2_ was low, even after PMA stimulation; however, a slight increase in H_2_O_2_ levels was observed in the presence of CTX. In the treated-fMLP groups, a significant and notable increase in H_2_O_2_ production by the THP-1 cells was observed when treated with CTX, both under baseline and PMA-stimulated conditions. Additionally, in the PMA-stimulated condition, the cells reached a plateau, showing no significant difference between non-silenced cells and fMLP-treated cells.

To further investigate the modulatory effect of CTX on FPR1 expression and the related signaling proteins in MϕFPRs^−/−^ cells, Western blotting was used to analyze protein levels. Our results showed that FPR1 expression was reduced in the absence of CTX treatment, confirming the effectiveness of our FPR silencing technique. However, CTX treatment in MϕFPRs^−/−^ cells demonstrated an increase in FPR1 expression suggesting that CTX stimulates the expression of both FPR1 and FPR2. Human FPR1 and FPR2 share approximately 69% amino acid sequence identity and activate common signaling pathways. Activation of FPRs on phagocytic cells activates Gi Protein resulting in the dissociation of the Gα and Gβγ subunits [34,44]. Subunit Gβγ activates downstream signaling pathways involving the PLCβ (phospholipase Cβ), PI3K (phophoinositide-3 kinase), and SKF (Src family kinase) families. In addition, GTPases from the Ras family, including Rho, Rac, and CDC42, are activated by the Gα subunit, which activates PI3K, leading to an accumulation of PIP3 (3’phosphatidyl-inositol-3,4,5-trisphosphate) at the phagocyte membrane, which is crucial for initiating chemotaxis [44]. PIP3 triggers a positive feedback loop involving Rac, which organizes actin filaments at the leading edge of the cells, facilitating cell movement and spreading toward chemoattractive signals [45]. Our results demonstrated that PI3K levels were very similar in the non-silenced and fMLP-stimulated cells. However, in the silenced cells, both the control and CTX-treated, PI3K expression was reduced, accompanied by a reduction in Rac levels in the same group. CTX did not modulate these proteins, which may explain the lack of inhibitory action on the spreading activity in the FPR-silenced group. These findings reinforce evidence that FPRs are essential for CTX action.

Additionally, another FPR downstream signaling pathway mediated by the Gβγ subunit involves the Src family kinase (SKF), which regulates chemotaxis and respiratory burst [46]. Our results demonstrated that in the MϕFPRs^−/−^ cells, Src expression was markedly decreased compared to the non-silenced and fMLP-stimulated cells. The reduction in Src expression was more pronounced in the presence of CTX. Conversely, in fMLP-stimulated cells treated with CTX, Src expression was elevated. These findings suggest a correlation between Src protein expression and H_2_O_2_ production.

The literature and our results in the THP-1 cells demonstrate that FPR plays a key role in the actions of the toxin, with differences in protein expression in some signaling molecules. However, using CTX conjugated with FITC, it was possible to observe the toxin entry in all THP-1 monocyte and THP-1-differentiated macrophage conditions, even in the cells where the FPRs were silenced. Boc-2 is widely used as antagonists of FPR1 and FPR2 [47,48]. In our study, different Boc-2 concentrations (50 µM or 100 µM) did not affect CTX entry into macrophages, suggesting that FPR-2 is not crucial for toxin internalization. To confirm this, macrophages obtained from rat peritoneum were treated with CTX which, as expected, inhibited phagocytosis activity. However, in the presence of Boc-2, CTX activity was abolished but the toxin still entered the cell. Corroborating these findings, previous studies demonstrated that pre-treatment with Boc-2 abolished the stimulatory effects of CTX on the secretory activity of macrophages co-cultivated with tumor cells [16]. These results demonstrate that CTX exerts its pharmacological effect in both FPR1-silenced cells and in cells treated with Boc-2, but its entry into macrophages is not related to these receptors. A recent study demonstrated that CTX was detected inside macrophages within 1 min of incubation and remained detectable after 24 h. The study also showed that CTX entry is facilitated through anionic lipids by inducing pore formation, which allows its internalization [49].

Therefore, complementary data aimed to understand the importance of G-protein-coupled receptors (GPCR) in CTX activity as well as the CA and CB subunits. An in silico screening was also performed to explore GPCR-related interactions with CTX subunits with the original research proposal, providing information to identify promising fragments for investigating the toxin’s functions and activities. Through DrugBank, molecules with structures similar to the CA and CB subunits of CTX were identified, providing information about potential molecular targets and their indications. Interestingly, some molecules exhibit biological activities similar to those observed for the toxin, known ligands of G-protein-coupled receptors (GPCRs), including FPR1, FPR2, and receptors involved in inflammation and immune signaling. Specific fragments showed structural affinity with modulators of pain, inflammation, and phagocytic activity pathways, similar to biological activity described to CTX [12,13,16,17]. This further suggests the potential molecular targets and unexplored functions of the toxin, especially those targeting GPCR associated with hormones, such as glucagon-like peptide 1 receptor (GLP-1R), melanocyte-stimulating hormone receptor (MC1-R), and gonadotropin-releasing hormone receptor (GnHR). It is worth mentioning that although we identified potential similarities, we did not perform docking simulations or functional validation. Therefore, the predictions mentioned here are preliminary and hypothesis-generating and allow us to understand which CTX fragments may interact with the receptors and trigger their biological effects.

## 5. Conclusions

Formyl peptide receptors play a crucial role in the immunomodulatory activities of CTX on macrophage functions, including ROS release, spreading, and phagocytosis. FPR1 silencing confirmed its involvement in these processes, reinforcing the importance of formyl peptide receptors in CTX-mediated effects. Additionally, it was observed that while CTX exerts its pharmacological effects in both FPR1-silenced cells and in cells treated with Boc-2, its entry into macrophages occurs independently of these receptors. Collectively, these findings support the notion that both FPR1 and FPR2 are essential for the immunomodulatory actions of CTX described thus far.

## Data Availability

All data are included in the manuscript.

## Supplementary Materials

The following supporting information can be downloaded at: https://www.mdpi.com/article/10.3390/cells14151159/s1, Figure S1: Differentiation of THP-1 cells with PMA inducing agent.; Table S1: Drugs similar to the α chain of the CA subunit.; Table S2: Drugs similar to the β chain of the CA subunit.; Table S3: Drugs similar to the γ chain of the CA subunit.; Table S4: Drugs similar to the CB subunit.; Table S5: Similarity of receivers between FPR and FPR1.
