# Supplementary material for: Formyl Peptide Receptors 1 and 2: Essential for Immunomodulation of Crotoxin in Human Macrophages, Unrelated to Cellular Entry
Source: Cells. 2025 Jul 26;14(15):1159. doi: 10.3390/cells14151159 (PMC12345708; doi:10.3390/cells14151159)
Supplement: Supplementary file 1 [file cells-14-01159-s001.zip › Figure 1S Legend.pdf-1.pdf]

Lagend:

Figure S1. Differentiation of THP-1 cells with PMA inducing agent. THP-1 cells ($1x10^6$) were incubated with RPMI-1640 culture medium and in the presence of the inducing agent Phorbol myristate acetate ester (PMA) in culture bottles for cell differentiation. **A** - THP-1 cells in their monocytic form in suspension (mϕFPRs$^{+/+}$). **B** - Differentiated THP-1 cells (MϕFPRs$^{-/-}$), adhered in the presence of only RPMI-1640 culture medium.
