# Supplementary material for: Formyl Peptide Receptors 1 and 2: Essential for Immunomodulation of Crotoxin in Human Macrophages, Unrelated to Cellular Entry
Source: Cells. 2025 Jul 26;14(15):1159. doi: 10.3390/cells14151159 (PMC12345708; doi:10.3390/cells14151159)
Supplement: Supplementary file 1 [file cells-14-01159-s001.zip › Table 1S.pdf]

Table 1S* - Drugs similar to the α chain of the CA subunit

CA - Chain α					
**Similarity**	Name	Chemical Formula	Groups	Targets	Indication
**Score: 0.902**	Semaglutide	$C_{187}H_{291}N_{45}O_{59}$	approved; investigational	Glucagon-like peptide 1 receptor	Indicated to improve glycemic control in adults with type 2 diabetes mellitus as an adjunct of diet and exercise.
**Score: 0.895**	CZEN 002	$C_{77}H_{109}N_{21}O_{19}S$	Investigational	Melanocyte-stimulating hormone receptor	Investigated for use/treatment in candidiasis and vaginitis.
**Score: 0.856**	Triptorelin	$C_{64}H_{82}N_{18}O_{13}$	approved; vet approved	Gonadotropin-releasing hormone receptor	Indicated for the palliative treatment of advanced prostate, breast and salivary gland cancer.
**Score: 0.82**	Aclerastide	$C_{42}H_{64}N_{12}O_{11}$	Investigational	Not Available	Investigated for the treatment of diabetic foot, diabetic foot ulcers, and foot ulcer, diabetic.
**Score: 0.805**	Angiotensin 1-7	$C_{41}H_{62}N_{12}O_{11}$	Investigational	Not Available	Bone Cancer / Chondrosarcomas / Clear Cell Sarcoma of the Kidney / Metastatic Osteosarcoma / Ovarian Sarcoma / Adult Soft Tissue Sarcoma / Uterine Sarcoma
**Score: 0.745**	Lutetium Lu 177 dotatate	$C_{65}H_{87}LuN_{14}O_{19}S_2$	approved; investigational	Somatostatin receptor types 1,2,3,4,5	Indicated for the treatment of somatostatin receptor-positive gastroenteropancreatic neuroendocrine tumors (GEP-NETs), including foregut, midgut, and hindgut neuroendocrine tumors in adults
**Score: 0.725**	Daptomycin	$C_{72}H_{101}N_{17}O_{26}$	approved; investigational	Bacterial outer membrane (Incorporation into and destabilization) / Lipoteichoic acid synthesis (Inhibitor)	For the treatment of complicated skin and skin structure infections caused by susceptible strains of Gram-positive microorganisms / Sepsis.

*Table transcribed in full as expressed in the DrugBank database
