# Supplementary material for: Formyl Peptide Receptors 1 and 2: Essential for Immunomodulation of Crotoxin in Human Macrophages, Unrelated to Cellular Entry
Source: Cells. 2025 Jul 26;14(15):1159. doi: 10.3390/cells14151159 (PMC12345708; doi:10.3390/cells14151159)
Supplement: Supplementary file 1 [file cells-14-01159-s001.zip › Table 2S.pdf]

Table 2S* - Drugs similar to the β chain of the CA subunit

CA - Chain β					
**Similarity**	Name	Chemical Formula	Groups	Targets	Indication
**Score: 0.806**	Bivalirudin	$C_{98}H_{138}N_{24}O_{33}$	approved; investigational	Prothrombin	Indicated for treatment of heparin-induced thrombocytopenia and for the prevention of thrombosis.
**Score: 0.853**	Ularitide	$C_{145}H_{234}N_{52}O_{44}S_3$	investigational	Atrial natriuretic peptide receptor 1	Investigated for use/treatment in congestive heart failure.
**Score: 0.802**	Corticorelin ovine triflutate	$C_{205}H_{339}N_{59}O_{63}S$	approved	Corticotropin - releasing factor receptor 1	Indicated for use in differentiating pituitary and ectopic production of ACTH in patients with ACTH-dependent Cushing's syndrome. / Pituitary neoplasms.
**Score: 0.74**	Cilengitide	$C_{27}H_{40}N_8O_7$	investigational	Not Available	Used in trials studying the treatment of sarcoma, gliomas, lymphoma, leukemia, and lung cancer, among others.
**Score: 0.719**	Atosiban	$C_{43}H_{67}N_{11}O_{12}S_2$	approved; investigational	Not Available	Atosiban is an inhibitor of the hormones oxytocin and vasopressin. It is used as an intravenous medication as a labour repressant (tocolytic) to halt premature labor.
**Score: 0.712**	Carbetocin	$C_{45}H_{69}N_{11}O_{12}S$	approved; investigational	Oxytocin receptor	Used to control postpartum hemorrhage and bleeding after giving birth.
**Score: 0.709**	T131	$C_{86}H_{140}N_{32}O_{18}S_2$	investigational	Peroxisome proliferator-activated receptor gamma	Investigated for use/treatment in diabetes mellitus type 2.
**Score: 0.705**	Tifuvirtide	$C_{235}H_{341}N_{57}O_{67}$	investigational	Plasma serine protease inhibitor	Investigated for use/treatment in acquired immune deficiency syndrome (AIDS) and aids-related infections and HIV infection.

*Table transcribed in full as expressed in the DrugBank database
