# Supplementary material for: Formyl Peptide Receptors 1 and 2: Essential for Immunomodulation of Crotoxin in Human Macrophages, Unrelated to Cellular Entry
Source: Cells. 2025 Jul 26;14(15):1159. doi: 10.3390/cells14151159 (PMC12345708; doi:10.3390/cells14151159)
Supplement: Supplementary file 1 [file cells-14-01159-s001.zip › Table 3S.pdf]

Table 3S* - Drugs similar to the γ chain of the CA subunit

CA - Chain γ					
**Similarity**	Name	Chemical Formula	Groups	Targets	Indication
**Score: 0.77**	Bradykinin	$C_{50}H_{73}N_{15}O_{11}$	investigational	Not Available	Investigated for the basic science and treatment of hypertension and diabetes type 2.
**Score: 0.765**	Bivalirudin	$C_{98}H_{138}N_{24}O_{33}$	approved; investigational	Prothrombin	Indicated for treatment of heparin-induced thrombocytopenia and for the prevention of thrombosis.
**Score: 0.736**	T131	$C_{86}H_{140}N_{32}O_{18}S_2$	investigational	Peroxisome proliferator-activated receptor gamma	Investigated for use/treatment in diabetes mellitus type 2.
**Score: 0.735**	Terlipressin	$C_{52}H_{74}N_{16}O_{15}S_2$	approved; investigational	Vasopressin V1a, V1b and V2 receptor	Commonly used to stop bleeding of varices in the food pipe (oesophagus).
**Score: 0.732**	Desmopressin	$C_{46}H_{64}N_{14}O_{12}S_2$	approved	Vasopressin V1a, V1b and V2 receptor	Indicated for the treatment of nocturia due to nocturnal polyuria/ Indicated as antidiuretic replacement therapy in the management of central cranial diabetes / Indicated for patients with hemophilia A with factor VIII coagulant activity levels greater than 5%
**Score: 0.729**	Atosiban	$C_{43}H_{67}N_{11}O_{12}S_2$	approved; investigational	Not Available	Atosiban is an inhibitor of the hormones oxytocin and vasopressin. It is used as an intravenous medication as a labour repressant (tocolytic) to halt premature labor.
**Score: 0.721**	Ularitide	$C_{145}H_{234}N_{52}O_{44}S_3$	investigational	Atrial natriuretic peptide receptor 1	Investigated for use/treatment in congestive heart failure.
**Score: 0.716**	Selepressin	$C_{46}H_{73}N_{13}O_{11}S_2$	investigational	Not Available	Used in trials studying the treatment of septic shock.

*Table transcribed in full as expressed in the DrugBank database
