# Supplementary material for: Formyl Peptide Receptors 1 and 2: Essential for Immunomodulation of Crotoxin in Human Macrophages, Unrelated to Cellular Entry
Source: Cells. 2025 Jul 26;14(15):1159. doi: 10.3390/cells14151159 (PMC12345708; doi:10.3390/cells14151159)
Supplement: Supplementary file 1 [file cells-14-01159-s001.zip › Table 4S.pdf]

Table 4S* - Drugs similar to the CB subunit

CB					
**Similarity**	**Name**	**Chemical Formula**	**Groups**	**Targets**	**Indication**
Score: 0.929	Tetracosactide	$C_{136}H_{210}N_{40}O_{31}S$	approved	Adrenocorticotropic hormone receptor	Used as a diagnostic agent in the screening of patients presumed to have adrenocortical insufficiency.
Score: 0.918	CZEN 002	$C_{77}H_{109}N_{21}O_{19}S$	investigational	Melanocyte-stimulating hormone receptor	Investigated for use/treatment in candidiasis and vaginitis.
Score: 0.876	Tifuvirtide	$C_{235}H_{341}N_{57}O_{67}$	investigational	Plasma serine protease inhibitor	Investigated for use/treatment in acquired immune deficiency syndrome (AIDS) and aids-related infections and HIV infection.
Score: 0.864	Afamelanotide	$C_{78}H_{111}N_{21}O_{19}$	approved; investigational	Melanocyte-stimulating hormone receptor	Investigated for use/treatment in actinic keratosis, keratoses, skin cell studies, and skin infections/disorders.
Score: 0.859	Semaglutide	$C_{187}H_{291}N_{45}O_{59}$	approved; investigational	Glucagon-like peptide 1 receptor	Indicated to improve glycemic control in adults with type 2 diabetes mellitus as an adjunct of diet and exercise.
Score: 0.854	Corticorelin ovine triflutate	$C_{205}H_{339}N_{59}O_{63}S$	approved	Corticotropin-releasing factor receptor 1	Indicated for use in differentiating pituitary and ectopic production of ACTH in patients with ACTH-dependent Cushing's syndrome. / Pituitary Neoplasms
Score: 0.807	Triptorelin	$C_{64}H_{82}N_{18}O_{13}$	approved; vet_approved	Gonadotropin-releasing hormone receptor	Indicated for the palliative treatment of advanced prostate cancer. / Breast Cancer / Salivary Gland Cancers
Score: 0.786	Aclerastide	$C_{42}H_{64}N_{12}O_{11}$	investigational	Not Available	Investigated for the treatment of Diabetic Foot, Diabetic Foot Ulcers, and Foot Ulcer, Diabetic.
Score: 0.784	Ularitide	$C_{145}H_{234}N_{52}O_{44}S_3$	investigational	Atrial natriuretic peptide receptor 1	Investigated for use/treatment in congestive heart failure.
Score: 0.783	Somatostatin	$C_{76}H_{104}N_{18}O_{19}S_2$	approved; investigational	Somatostatin receptor types 1, 2, 3, 4, 5	Indicated for the symptomatic treatment of acute bleeding from esophageal varices.
Score: 0.775	Lanreotide	$C_{54}H_{69}N_{11}O_{10}S_2$	approved	Somatostatin receptor type 2 / type 5	Indicated for treatment of neuroendocrine tumours and acromegaly.
Score: 0.771	Angiotensin 1-7	$C_{41}H_{62}N_{12}O_{11}$	investigational	Not Available	Bone Cancer / Chondrosarcomas / Clear Cell Sarcoma of the Kidney / Metastatic Osteosarcoma / Ovarian Sarcoma / Adult Soft Tissue Sarcoma / Uterine Sarcoma
Score: 0.768	Pentagastrin	$C_{37}H_{49}N_7O_9S$	approved	Gastrin/cholecystokinin type B receptor	Used as a diagnostic aid for evaluation of gastric acid secretory function
Score: 0.762	Tesamorelin	$C_{216}H_{360}N_{72}O_{63}S$	approved; investigational	Growth hormone-releasing hormone receptor	Tesamorelin acetate is a synthetic analogue of human hypothalamic Growth Hormone Releasing Factor (hGRF) indicated to induce and maintain a reduction of excess abdominal fat in HIV-infected patients with lipodystrophy.
Score: 0.762	TAK-448	$C_{58}H_{80}N_{16}O_{14}$	investigational	Not Available	Used in trials studying the treatment of Prostate Cancer, Low Testosterone, Prostatic Neoplasms, and Hypogonadotropic Hypogonadism
Score: 0.755	TT-232	$C_{45}H_{58}N_{10}O_9S_2$	investigational	Not Available	Tln 232 is under investigation in clinical trial NCT00422786 (Phase II Study of CAP-232 in Patients With Refractory Metastatic Renal Cell Carcinoma)
Score: 0.749	BQ-123	$C_{31}H_{42}N_6O_7$	investigational	Not Available	Investigated for the basic science and treatment of Coronary Artery Disease, Aorto-coronary Bypass Grafting, and ST-Elevation Myocardial Infarction
Score: 0.74	Pentetreotide	$C_{63}H_{87}N_{13}O_{19}S_2$	approved; investigational	Not Available	Pentetreotide has been used in trials studying the diagnostic of cushing syndrome.
Score: 0.739	Daptomycin	$C_{72}H_{101}N_{17}O_{26}$	approved; investigational	Bacterial outer membrane (Incorporation into and destabilization)/Lipoteichoic acid synthesis (Inhibitor)	Indicated for the treatment of complicated skin and skin structure infections caused by susceptible strains of Gram-positive microorganisms. / Sepsis
Score: 0.733	Bivalirudin	$C_{98}H_{138}N_{24}O_{33}$	approved; investigational	Prothrombin	Indicated for treatment of heparin-induced thrombocytopenia and for the prevention of thrombosis.
Score: 0.73	Ularitide	$C_{145}H_{234}N_{52}O_{44}S_3$	investigational	Atrial natriuretic peptide receptor 1	Investigated for use/treatment in congestive heart failure.
Score: 0.73	Lixisenatide	$C_{215}H_{347}N_{61}O_{65}SV$	approved	Glucagon-like peptide 1 receptor	Used as an antihyperglycemic agent in the treatment of T2DM.
Score: 0.73	Setmelanotide	$C_{49}H_{68}N_{18}O_9S_2$	investigational	Not Available	Investigated for the treatment of Obese and Overweight.
Score: 0.728	Octreotide	$C_{49}H_{66}N_{10}O_{10}S_2$	approved; investigational	Somatostatin receptor type 1 / type 5/ type 2	Indicated for treatment of acromegaly and reduction of side effects from cancer chemotherapy
Score: 0.725	Somatoprim	$C_{60}H_{74}N_{12}O_{10}$	investigational	Not Available	Somatoprim is under investigation for the treatment of Acromegaly.

*Table transcribed in full as expressed in the DrugBank database
