# Supplementary material for: Formyl Peptide Receptors 1 and 2: Essential for Immunomodulation of Crotoxin in Human Macrophages, Unrelated to Cellular Entry
Source: Cells. 2025 Jul 26;14(15):1159. doi: 10.3390/cells14151159 (PMC12345708; doi:10.3390/cells14151159)
Supplement: Supplementary file 1 [file cells-14-01159-s001.zip › Table 5S.pdf]

Table 5S* - Similarity of receivers between FPR and FPR1

| Target Name | E-value (FPR) | E-value (FPR1) |
|---|---|---|
| FMLP receptor | 0.0 | 4e-176 |
| FMLP-related receptor I | 4e-176 | 0.0 |
| Glucagon-like peptide 1 receptor | - | - |
| Melanocyte-stimulating hormone receptor | 0.007 | 0.14 |
| Gonadotropin-releasing hormone receptor | 0.008 | 7e-06 |
| Somatostatin receptor type 1 | 2e-40 | 6e-39 |
| Somatostatin receptor type 2 | 3e-40 | 2e-35 |
| Somatostatin receptor type 3 | 6e-22 | 2e-25 |
| Somatostatin receptor type 4 | 3e-35 | 1e-32 |
| Somatostatin receptor type 5 | 2e-30 | 1e-31 |
| Prothrombin | - | - |
| Atrial natriuretic peptide receptor 1 | - | - |
| Corticotropin - releasing factor receptor 1 | - | - |
| Oxytocin receptor | 7e-09 | 2e-11 |
| Peroxisome proliferator-activated receptor gamma | - | - |
| Plasma serine protease inhibitor | - | - |
| Vasopressin V1a receptor | 4e-12 | 1e-16 |
| Vasopressin V1b receptor | 4e-07 | 5e-10 |
| Vasopressin V2 receptor | 4e-06 | 9e-09 |
| Adrenocorticotropic hormone receptor | 0.35 | 0.26 |
| Gastrin/cholecystokinin type B receptor | 2e-13 | 1e-11 |
| Growth hormone-releasing hormone receptor | - | - |

*Table fully transcribed as expressed in the TTD database
