# Supplementary figures and images for: Formyl Peptide Receptors 1 and 2: Essential for Immunomodulation of Crotoxin in Human Macrophages, Unrelated to Cellular Entry
Source: Cells. 2025 Jul 26;14(15):1159. doi: 10.3390/cells14151159 (PMC12345708; doi:10.3390/cells14151159)

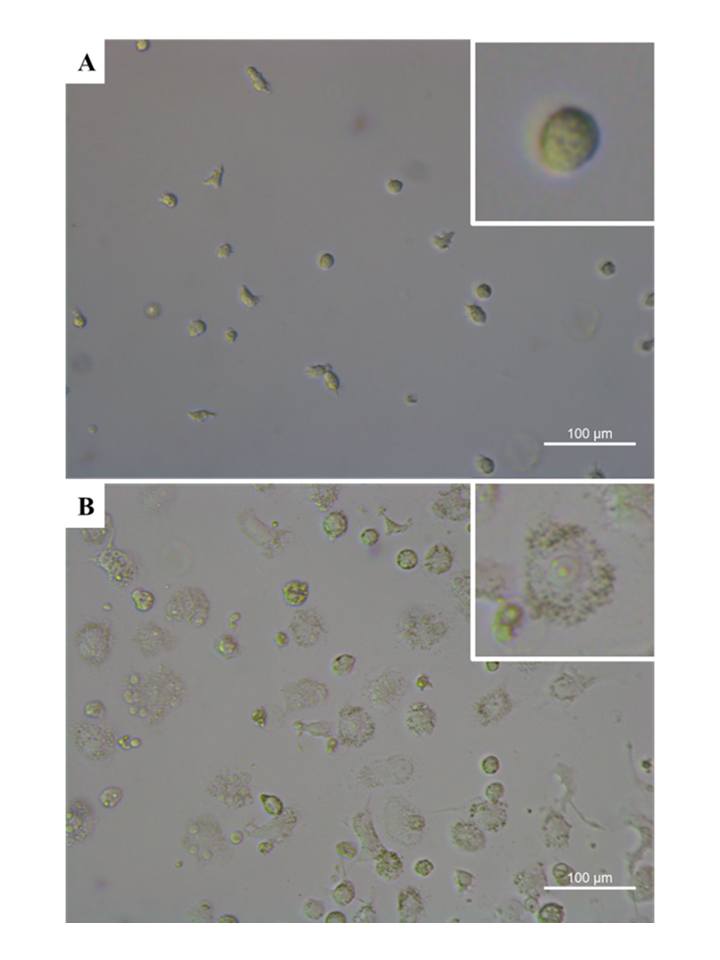

Supplement: Supplementary file 1 [file cells-14-01159-s001.zip › Figure 1S.tif]
